# Automatic Detection of Gait Perturbations With Everyday Wearable Technology

**DOI:** 10.1109/OJEMB.2025.3624591

**Published:** 2025-10-23

**Authors:** L. Feld, S. Hellmers, L. Schell-Majoor, J. Koschate-Storm, T. Zieschang, A. Hein, B. Kollmeier

**Affiliations:** ^1^ Department for Health Services Research, Assistance Systems and Medical Device TechnologyCarl von Ossietzky University 11233 26129 Oldenburg Germany; ^2^ Department of Medical Physics and Acoustics, Medical Physics and Cluster of Excellence Hearing4allCarl von Ossietzky University 11233 26129 Oldenburg Germany; ^3^ Department for Health Services Research, Geriatric MedicineCarl von Ossietzky University 11233 26129 Oldenburg Germany

**Keywords:** Fall risk monitoring, gait perturbation detection, wearable sensors, hearables, hearing aids

## Abstract

*Objective:* Older adults face a heightened fall risk, which can severely impact their health. Individual responses to unexpected gait perturbations (e.g., slips) are potential predictors of this risk. This study examines automatic detection of treadmill-generated gait perturbations using acceleration and angular velocity from everyday wearables. Detection is achieved using a deep convolutional long short-term memory (DeepConvLSTM) algorithm. *Results:* An F1 score of at least 0.68 and recall of 0.86 was retrieved for all data, i.e., data from hearing aids, smartphones at various positions and professional sensors at lumbar and sternum. Performance did not significantly change when combining data from different sensor positions or using only acceleration data. *Conclusion:* Results suggest that hearing aids and smartphones can monitor gait perturbations with similar performance as professional equipment, highlighting the potential of everyday wearables for continuous fall risk monitoring.

## I. Introduction

Falls represent a critical event in the lives of older adults, often leading to significant negative consequences such as concerns about falling, reduced mobility, and decreased independence, even after falls without physical injuries [1]. Consequently, preventing falls is essential for maintaining the quality of life of this population [2], [3]. Several factors increase fall risk [4], including poor reactive balance to near-falls and gait perturbations like slips, trips, or missteps, which often precede falls [5], [6], [7], [8].

Continuous daily monitoring could help predict falls, which draws research attention to the use of inertial measurement units (IMUs). Existing studies [7], [9], [10], [11], [12] examined the detection of near-falls and perturbations using IMUs, typically placed at the waist or chest. Extending beyond these placements, one study [13] demonstrated that near-falls induced during standing can also be detected using a wrist-worn smartwatch. Additionally, smartphones and ear-worn sensors have attracted growing interest for motion and balance monitoring, with studies exploring their potential for gait analysis [14], [15], [16], [17], [18] and fall detection [19], [20], [21]. However, to our knowledge there are no studies till now that examine the automatic detection of near-falls with smartphones and hearing aids at their typical wearing positions. These devices seem to offer great potential for continuous monitoring as they are commonly worn in everyday life and often already contain IMUs.

In this manuscript the automatic detection of gait perturbations with everyday wearable technology utilizing a deep convolutional long short-term memory (DeepConvLSTM) algorithm is investigated. Several research questions are addressed. First, a comparison between data recorded at usually used sensor positions (i.e., standard positions), where the sensors are placed near the center of mass, and uncommon wearing positions is investigated. This consideration leads to the question:

1) Are smartphones and hearing aids able to detect gait perturbations as well as professional equipment, placed at standard positions like the lumbar or sternum region?

It is also investigated if the use of one sensor is sufficient, offering a more practical solution for real-life applications. This consideration leads to the question:

2) Does combining data from everyday wearable technologies at their typical wearing positions give substantial benefit?

Several studies have shown that tasks such as gait classification [22], fall detection [23], and activity recognition [24] can be effectively performed using only accelerometer data, even just with two axes of it [25]. This leads to the last research question:

3) How does performance of the algorithm change when trained and tested only with acceleration data?

## II. Results

To evaluate the performance of the DeepConvLSTM algorithm, precision, recall and F1 score are used. Precision is the proportion of correctly classified perturbation events among all events classified as perturbations. Recall is the proportion of correctly classified perturbation events among all actual perturbation events. The F1 score is the harmonic mean of precision and recall.

### A. Comparison of Sensor Positions

The performance metrics for all sensor positions are depicted in Table 1. The algorithm showed at least a precision of 0.57, a recall of 0.86 and an F1 score of 0.68 for the data from each position. For all positions, precision was lower than recall. The one-way repeated measures ANOVA showed a significant effect for sensor position on the F1 score ($p$<0.001, partial $\eta ^{2}$ = 0.599). Post-hoc comparisons indicated no significant difference in F1 score (around 0.83) was observed for data recorded with the professional sensor at the lumbar (L) position, as well as data collected with the hearing aids (LHA, RHA). In contrast, the F1 scores for these data were significantly higher than those for data recorded with smartphones in the jacket pocket, pants pocket, and shoulder bag (JP, PP, SB). Additionally, the F1 score for data recorded with the professional sensor at the sternum (S) position was significantly lower than for the right hearing aid (RHA). Notably, the F1 score showed higher variability for the pants pocket and shoulder bag conditions compared to all other conditions.

**TABLE 1:** Precision, Recall, and F1 Score for the DeepConvLSTM Algorithm are Provided for Data Recorded With Professional Equipment At Standard Positions (Lumbar and Sternum) and Everyday Wearable Technology At Typical Wearing Positions (LHA/RHA: Left/right Hearing Aid, JP/PP/SB: Smartphone in a Jacket pocket/pants pocket/shoulder Bag). Performance Metrics (Mean $\pm$ Standard Deviation) Were Calculated on Test Sets From 10 Random Participant-Level Splits. The F1 Scores for Data Obtained From the Lumbar, RHA, and LHA Sensors Were Significantly Higher Than Those From the Smartphone Placed at the JP and PP Positions. Additionally, the F1 Score for Data Obtained From the Smartphone in the SB Position was Significantly Lower Than That From the LHA. The F1 Score for Data Obtained From the Sternum Position was Also Significantly Lower Compared to Those From the RHA and LHA.

**trained and tested with**	**precision**	**recall**	**F1**
Lumbar	$0.72\pm 0.04$	$0.92\pm 0.02$	$0.81\pm 0.03$
Sternum	$0.64\pm 0.12$	$0.91\pm 0.02$	$0.75\pm 0.07$ ^a^
RHA	$0.82\pm 0.05$	$0.91\pm 0.05$	$0.86\pm 0.03$
LHA	$0.79\pm 0.05$	$0.93\pm 0.06$	$0.85\pm 0.03$
JP	$0.59\pm 0.10$	$0.86\pm 0.05$	$0.70\pm 0.03$ ^b^
PP	$0.57\pm 0.10$	$0.89\pm 0.04$	$0.68\pm 0.07$ ^b^
SB	$0.59\pm 0.14$	$0.88\pm 0.03$	$0.70\pm 0.10$ ^c^

^0^ ^a^ significant different to positions RHA and LHA ($p < 0.05$)

^0^ ^b^ significant different to positions Lumbar, RHA and LHA ($p < 0.05$)

^0^ ^c^ significant different to position LHA ($p < 0.05$)

### B. Combination of Sensor Positions

Table 2 shows the performance metrics for the combination of data recorded with everyday wearable technologies at their typical wearing positions. The analyses focused on combinations that are more commonly found in everyday life. These includes using data from two hearing aids as well as combining data from one hearing aid with data from a smartphone, instead of using data from two smartphones. The Friedmann test showed no significant effect for sensor position combinations on the F1 score ($p$ = 0.634, $\chi ^{2}$ = 2.56).

**TABLE 2:** Precision, Recall, and F1 Score for the DeepConvLSTM Algorithm for the Combination of Data Recorded With Everyday Wearable Technology At Typical Wearing Positions (LHA/RHA: Left/right Hearing Aid, JP/PP/SB: Smartphone in a Jacket pocket/pants pocket/shoulder Bag). Performance Metrics (Mean $\pm$ Standard Deviation) Were Calculated on Test Sets From 10 Random Participant-Level Splits.

**trained and tested with**	**precision**	**recall**	**F1**
LHA+RHA	$0.75\pm 0.07$	$0.91\pm 0.06$	$0.82\pm 0.05$
LHA+JP	$0.78\pm 0.10$	$0.90\pm 0.04$	$0.83\pm 0.06$
LHA+SB	$0.76\pm 0.12$	$0.92\pm 0.05$	$0.83\pm 0.08$
LHA+PP	$0.82\pm 0.08$	$0.91\pm 0.05$	$0.86\pm 0.05$

### C. Performance for Acceleration Data Only

Table 3 presents the performance of the detection algorithm when using only acceleration data, instead of the combined use of acceleration and gyroscope data (see Table 1). For clarity, the analyses was limited to three sensor positions, the lumbar position and the hearing aids. These positions were selected because, among the standard placements, the lumbar outperformed the sternum, and the hearing aids showed substantially better performance than the smartphones. The two-way repeated measures ANOVA was conducted to evaluate the effect of sensor type (accelerometer only vs. accelerometer and gyroscope) on the F1 scores. The main effect for sensor type was not significant ($p=0.665$, partial $\eta ^{2}=0.022$), indicating that the inclusion of gyroscope data did not improve performance.

**TABLE 3:** Precision, Recall, and F1 Score for the DeepConvLSTM Algorithm for Acceleration Data Recorded With Professional Equipment At the Lumbar and Everyday Wearable Technology At the Ear (LHA/RHA: Left/right Hearing Aid). Performance Metrics (Mean $\pm$ Standard Deviation) Were Calculated on Test Sets From 10 Random Participant-Level Splits.

**trained and tested with**	**precision**	**recall**	**F1**
Lumbar$_{accel.}$	$0.73\pm 0.11$	$0.93\pm 0.03$	$0.81\pm 0.08$
RHA$_{accel.}$	$ 0.80\pm 0.06$	$0.91\pm 0.054$	$0.85\pm 0.05$
LHA$_{accel.}$	$0.82\pm 0.05$	$0.93\pm 0.03$	$0.87\pm 0.04$

## III. Discussion

It should be noted that the high inter-individual variability, especially given the broad age range of participants, could limit the algorithm's performance. Previous research shows that results from younger adults often do not generalize to older adults or those at increased fall risk (e.g. [26], [27], [28]). Future studies should focus on older populations with elevated fall risk to develop more applicable and generalizable models.

### A. Comparison of Sensor Positions

For all sensor positions, the precision is lower than the recall, indicating that the algorithm is more likely to detect a perturbation when there is none (false positive) than to miss an actual perturbation (false negative). When considering the application of automatic gait perturbation detection for long-term monitoring, a higher recall is preferable to a higher precision. In long-term monitoring, it is crucial to detect as many of the perturbations that have taken place as possible to analyze the participants' reaction and identify changes early. This ensures timely recognition of potential declines in motor control or increased fall risk, enabling early intervention.

Comparing the neural network's performance across data collected at different sensor positions, hearing aids performed similar to the standard position lumbar. This aligns well with expectations based on the results of our previous study [29], which reported strong correlations for data collected at this position. This result also may reflect that head and trunk segments are stabilized with respect to the environment during walking [30], [31]. The results also align well with the results of Aziz et al. [32], where the head position showed similar performance than the waist for distinguishing near-falls from daily activities using a support vector machine. Among the smartphone positions, the data from the jacket pocket lead to the lowest variability. This might have been expected as the smartphone is attached to the torso, and has less way to manouver. The result also aligns well with the findings of Silsupadol et al. [33], which found that a smartphone in the pants pocket is less effective than one attached to the lumbar region for accurately estimating gait parameters. The high variability of the F1 score for data from the shoulder bag position may be due to the bag's movement, which was observed to vary with participants' stature. Additionally, the larger size of the bag compared to the smartphone allowed more movement of the smartphone in the bag.

The findings suggest that hearing aids should be able to detect gait perturbations with performance similar to professional equipment at standard positions like the lumbar or sternum. Hereby, a big advantage of hearing aids is their consistent placement throughout the day, unlike smartphones, which are carried in various positions, potentially complicating detection.

### B. Combination of Sensor Positions

Since no significant difference was found, combining two hearing aids does not appear to offer an advantage over using just one. This aligns well with our previous findings, where we observed a very strong correlation between the data from the two hearing aids (see [29]).

The combination of a smartphone and a hearing aid also shows no advantage. Similarly, Aziz et al. [32] found no performance gain when combining a head sensor with a sensor on the waist or sternum for distinguishing near-falls from daily activities using a support vector machine. This suggests that the information content of the data recorded at the head and the smartphone positions is not complementary enough to achieve an improvement. The results indicate that a single hearing aid provides comparable performance to the combination with a smartphone or a second hearing aid. This is promising, as minimizing the number of sensors is desirable for daily use.

### C. Performance for Acceleration Data Only

The results indicate that using only accelerometer data appears sufficient for the automatic detection of gait perturbations. Relying solely on accelerometer data can reduce power consumption and data storage requirements, which are important factors for wearable devices and long-term monitoring applications. Future research should explore whether data dimensionality can be further reduced. It is also important to verify with real-life perturbation data if accelerometer data alone remains sufficient, particularly for distinguishing between perturbations and activities of daily living.

## IV. Conclusion

In conclusion, the deep convolutional long short-term memory (DeepConvLSTM) algorithm performed comparably for data recorded from hearing aids and smartphones in various positions (jacket pocket, shoulder bag, pants pocket) to data recorded at standard positions (sternum, lumbar). Hearing aids, due to their consistency in placement and daily wearability, show great potential for head-based monitoring of gait perturbations, enabling an early detection of an increased fall risk. The results also indicate that a single device may be sufficient for reliable detection, which highlights the feasibility of everyday wearables, like smartphones and hearing aids, as practical tools for gait monitoring. Furthermore, the results suggest that it is sufficient to use only acceleration data.

The study demonstrates that the automatic detection of gait perturbations can achieve comparable performance using both professional equipment at standard positions and everyday wearable technology at typical wearing positions. Future work should incorporate real-life data, and investigate systematically other algorithms that may offer even better performance.

## V. Materials and Methods

This study was approved by the medical ethics committee of the University of Oldenburg (number 2021-093).

### A. Participants

Participants were recruited through flyers, a digital university blackboard, personal contacts, and an internal database. All participants provided written informed consent and received hourly financial compensation for their voluntary participation. Exclusion criteria included acute illnesses requiring medical monitoring, inability to provide informed consent, or inability to walk unaided for at least 20 minutes. Additionally, individuals over two meters tall, weighing more than 135 kg, or with a waist circumference exceeding 120 cm were excluded due to technical constraints.

The study included 66 adults (46 females, 20 males; mean age: 57.8 years [range: 18–87 years]). Participants had varied conditions; for example, one had a lumbar vertebral fracture, four had spinal stenosis, and six had herniated discs, while others had no gait-related conditions.

### B. Experimental Setup

The experiments were conducted in a controlled laboratory environment, where participants completed different walking tasks. Preferred walking speed was first determined on the lab floor. Participants then began walking on a perturbation treadmill (MGait, Motek Medical B.V., Amsterdam, the Netherlands; sampling frequency $f_{\mathrm{T}}=300$ Hz) at 50% of their selected speed, which was gradually increased to establish their preferred treadmill speed. After familiarization, participants completed a one-minute “gait trial” on the treadmill. Afterwards, nine different perturbations were introduced with the perturbation treadmill in a pseudo-randomized order with intervals of 20 to 30 seconds between each perturbation. This is referred to as a “perturbation trial”. Each participant underwent three perturbation trials. A detailed description of the perturbations can be found in Supplementary Materials C.

### C. Measurement Devices

**Figure 1.**
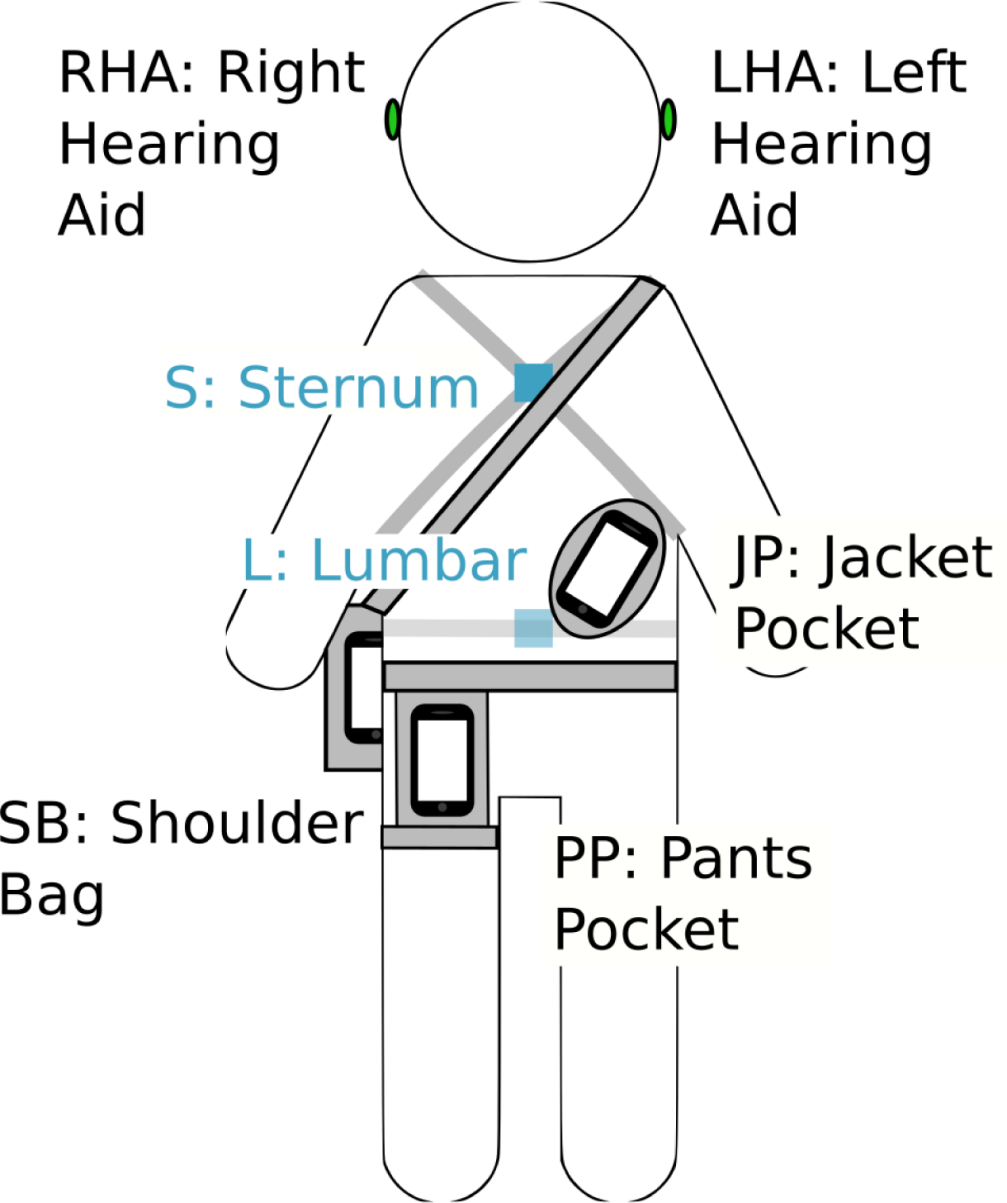
Measurement devices and their attachment positions: Professional equipment was positioned at lumbar and sternum level (blue). As everyday wearable technology (black), hearing aids were placed on the left and right ear, and smartphones were carried in a jacket pocket, shoulder bag, or pants pocket.

The acceleration and angular velocity of the participants' body was captured with different measurement devices at different attachment positions, as depicted in Fig. 1. As everyday wearable technologies three smartphones (Samsung Galaxy A52; sampling frequency $f_{\text{SP}}$ = 100Hz) and two hearing aids (Starkey Livio AI 2400; sampling frequency $f_{\text{HA}} = 104 \pm 4$ Hz; used solely as measurement device) were used. The hearing aids covered the position of the right (RHA) and left ear (LHA). The smartphones where placed at a jacket pocket (JP), pants pocket (PP), and a shoulder bag (SB). For comparison, professional equipment (Opal V1, Mobility Lab™(ML), APDM, Inc., Portland, OR, USA; sampling frequency $f_ {\text{APDM}}$ = 128 Hz) was used at different positions. Although our study included additional positions, this manuscript focuses exclusively on the lumbar (L) and sternum (S) levels for the placement of the professional equipment.

### D. Extraction of Gait and Perturbation Windows

From the synchronized data two-second windows were extracted and automatically labeled as “gait” or “perturbation”, depending on whether they came from the “gait trial” or “perturbation trial”. For the “gait windows”, two-second windows were cut out of the one-minute “gait trial” data using the sliding window method. The “perturbation windows” capture the participants' reactions to the perturbations from the “perturbation trial”. First, the time range of each perturbation, as marked by the treadmill, was identified, from start to end. Within this time range, the maximum value of the sensor's acceleration norm and the corresponding time point were determined. The “perturbation window” was then centered around this time point, with a duration of two seconds.

### E. Automatic Detection of Gait Perturbations

**Figure 2.**
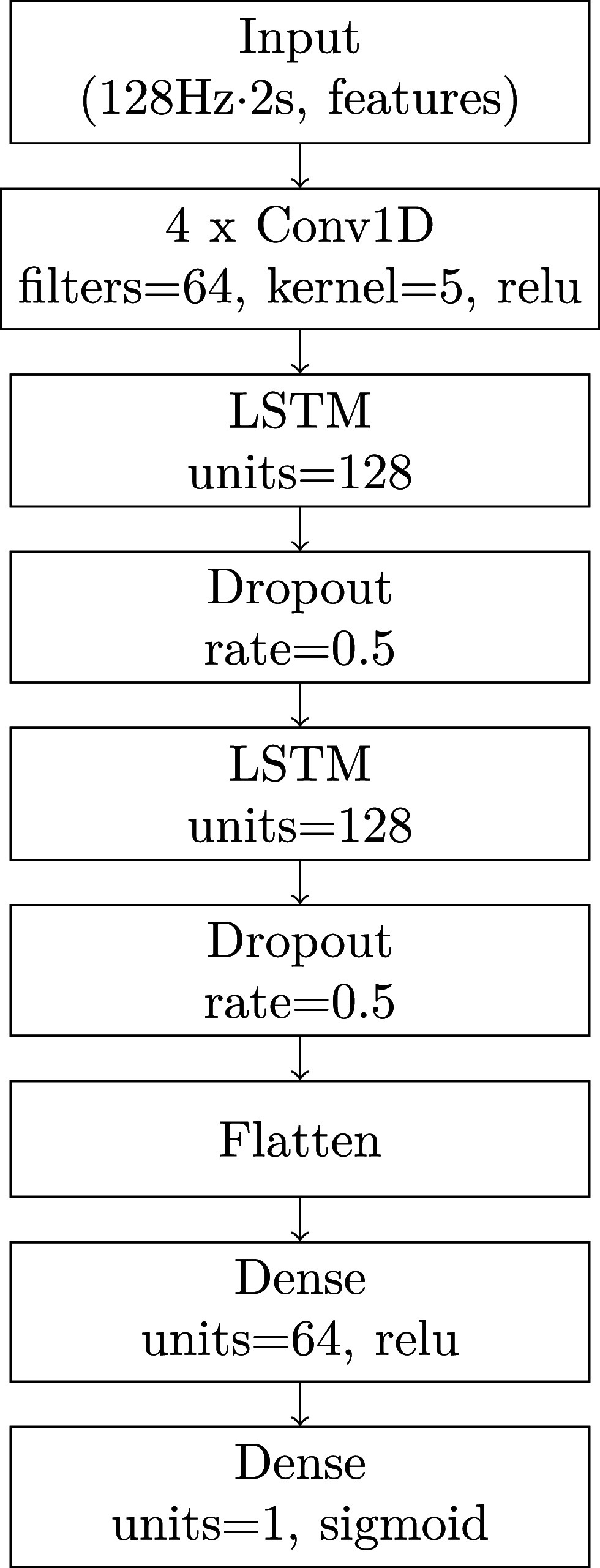
Architecture of the used DeepConvLSTM (Deep convolutional long short-term memory) network.

Despite its lower interpretability, a neural network was chosen over classical models because prior work [34] showed that classical models performed poorly on lumbar and sternum data (F1$\approx$0.4), while neural networks achieved higher scores. In a preliminary analysis (see Supplementary Materials A), the performance of the three neural networks proposed in that study were evaluated on lumbar data, optimizing their hyperparameters using hyperband. Based on this analysis, the deep convolutional long short-term memory (DeepConvLSTM) algorithm, which showed the best results, was selected for the analyses presented in this manuscript. The primary aim of this study was to compare sensor positions rather than to optimize the algorithm or pursue on-device deployment. Fig. 2 shows the architecture of the algorithm. The first part of the algorithm consists of four consecutive 1D convolutional layers, each using 64 filters and a kernel size of 5. The second part consists of two LSTM layers, each with 128 units. To prevent overfitting, dropout regularization with a rate of 0.50 is applied after the LSTM layers. The output of the final dropout layer is flattened and fed into a dense layer with 64 units and ReLU activation. The network ends with a single neuron using a sigmoid activation for binary classification. The network architecture was created using tensorflow and keras modules.

For each position, 10 random splits of the dataset into training (70%; $n$ = 46), validation (15%; $n$ = 10) and test (15%; $n$ = 10) data were created on participant level. Models were trained, validated and tested separately for each split. During training, the model uses class weights (computed with sklearn's $compute\_{c}lass\_{w}eight$ in balanced mode). Performance metrics (mean $\pm$ standard deviation) were calculated as average performance over all splits. Normality of the F1 scores for each sensor position and their combinations was evaluated using the Shapiro-Wilk test. The sensor positions were then compared using a one-way ANOVA. Due to violation of normal distribution, the combination of sensor positions were compared using a Friedman test. A two-way repeated measures ANOVA was conducted on the F1 scores for the factors sensor type and sensor position. If Mauchly's test showed that sphericity was violated, the Huynh-Feldt correction was applied. Post hoc comparisons were performed using Bonferroni-adjusted pairwise tests. Level of significance was set to p$\leq$0.05. Statistical analyses were performed using SPSS (version 29.0.0.0). The training and test process is explained in detail in [34].

## Supplementary Materials

The supplementary material includes a preliminary analysis, to evaluate the performance of different algorithms for the automatic detection of gait perturbations. Additionally the supplemental material contains additional analysis including: incorporation of data for additional normal walking conditions (that are more realistic than treadmill walking), training and testing the algorithm with data form different sensor positions, as well as the model performance for different perturbation types. Statistical details for the comparison of sensor positions shown in Table I are also included.

Supplementary Materials
