# Supplementary material for: Automatic Detection of Gait Perturbations With Everyday Wearable Technology
Source: IEEE Open J Eng Med Biol. 2025 Oct 23;6:570–5. doi: 10.1109/OJEMB.2025.3624591 (PMC12599889; doi:10.1109/OJEMB.2025.3624591)
Supplement: Supplementary Materials [file supp1-3624591.pdf]

Supplementary Materials

Automatic detection of gait perturbations with everyday wearable technology

L. Feld, S. Hellmers, L. Schell-Majoor, J. Koschate-Storm, T. Zieschang, A. Hein and B. Kollmeier

I. Comparison between different algorithms

In a preliminary analysis, the performance of the three algorithms proposed in [1] was evaluated on lumbar data, optimizing their hyperparameters using hyperband. Table I shows the optimized hyperparameters for a two-dimensional convolutional neural network (CNN), a one-dimensional convolutional neural network (1D-CNN) and a deep convolutional long short-term memory algorithm (DeepConvLSTM).

For all the algorithms window overlap was find to be not optimal. Window overlap indicates whether the consecutive two-second windows that were cut out of the data were generated with overlapping segments. Data augmentation was found to be optimal for the 1D-CNN and was performed after the idea of Um et al. [2]. New training data was generated by adding noise to the existing data, then segmenting the current data window into fixed blocks and shuffling them randomly. This process was repeated until the class ratio in the training data was balanced. Class imbalance was addressed for all algorithms using *sklearn.utils.class_weight*, which assigns higher weights to underrepresented classes. L2 regularization applied to the convolutional layers of the 1D-CNN adds a penalty term to the loss function, proportional to the squared magnitude of the model's weights to prevent overfitting The CNN consists of three two-dimensional convolutional layers (160, 64, 64), each with a kernel size of ($7x7$). The 1D-CNN consists of three convolutional layers (96, 128, 64), each with a kernel size of 5. The DeepConvLSTM algorithm combines convolutional and long short-term memory (LSTM) layers. The first part of the model processes the input sequence with four consecutive one-dimensional convolutional layers, each using 64 filters and a kernel size of five. The second part consists of two LSTM layers, each with 128 units. To reduce overfitting and improve generalization, dropout was applied to each algorithm. This technique randomly deactivates a fraction of neurons during training, preventing the model from relying too heavily on specific features. A dropout rate of 0.20 for the CNN and 1D-CNN and 0.50 for the DeepConvLSTM was found to be optimal. All algorithms were trained using a learning rate of 0.001, which controls the step size during weight updates.

Additionally to the hyperparameters, the performance, i.e., precision, recall, and F1 score, of each algorithm using acceleration and angular velocity data recorded at the lumbar level is shown in Table I. The CNN demonstrated the highest precision of 0.87 ± 0.09, but its recall was relatively low with 0.38 ± 0.12, resulting in a modest F1 score of 0.51 ± 0.11. In contrast, the 1D-CNN achieved a precision of 0.78 ± 0.08, a recall of 0.73 ± 0.09, and an F1 score of 0.75 ± 0.08 for lumbar-level data. The DeepConvLSTM outperformed the other tested models with the highest recall of 0.84 ± 0.07, a precision of 0.72 ± 0.07, and the best F1 score of 0.77 ± 0.04. Notably, the DeepConvLSTM also showed the lowest standard deviation, indicating greater consistency. Based on this analysis, the deep convolutional long short-term memory (DeepConvLSTM) algorithm, which showed the best results, was selected for the analyses presented in the main manuscript.

TABLE I. Optimized hyperparameters for a CNN, 1D-CNN and a DeepConvLSTM network as well as their performance (precision, recall and F1 score) for automatic gait perturbation classification with acceleration and angular velocity data measured at the lumbar level.

	CNN	1D-CNN	DeepConvL-STM
window overlap	False	False	False
data augmentation	False	True	False
class weights	True	True	True
L2 regularization	False	True	False
hidden layer 1	160 (Conv2D)	96 (Conv1D)	64 (4xConv1D)
hidden layer 2	64 (Conv2D)	128 (Conv1D)	128 (2xLSTM)
hidden layer 3	64 (Conv2D)	64 (Conv1D)	
kernel size	(7x7)	5	5
batch size	1024	128	128
dropout	0.20	0.20	0.50
lernrate	0.001	0.001	0.001
precision	0.87 ± 0.09	0.78 ± 0.08	0.77 ± 0.07
recall	0.38 ± 0.12	0.73 ± 0.09	0.91 ± 0.06
F1	0.51 ± 0.11	0.75 ± 0.08	0.83 ± 0.03

II. Additional analyses

A. Incorporation of different walking conditions

The main manuscript has focused solely on data collected under controlled treadmill conditions. However, the long-term goal is to extend the automatic detection algorithm to real-world, everyday scenarios. To initiate this transition, gait data from additional, more realistic walking conditions were incorporated alongside the previously used treadmill gait data.

In addition to the treadmill conditions, participants underwent an ”even” and ”uneven” walking condition. They walked six meters back and forth at their preferred speed on a flat floor for the ”even” and on structured surface plates with an additional staircase element (depicted in Fig. 1) for the ”uneven” walking condition. Both conditions were performed twice. Initially, they walked without head

treadmill data plus	Lumbar			LHA		
	Precision	Recall	F1	Precision	Recall	F1
even	0.72 ± 0.07	0.93 ± 0.03	0.81 ± 0.04	0.77 ± 0.06	0.93 ± 0.02	0.84 ± 0.04
even + even with head movement	0.74 ± 0.07	0.93 ± 0.03	0.83 ± 0.04	0.80 ± 0.06	0.90 ± 0.06	0.84 ± 0.03
uneven	0.73 ± 0.05	0.92 ± 0.03	0.81 ± 0.03	0.77 ± 0.04	0.94 ± 0.02	0.84 ± 0.03
uneven + uneven with head movement	0.76 ± 0.06	0.93 ± 0.02	0.83 ± 0.04	0.79 ± 0.05	0.92 ± 0.03	0.85 ± 0.03

TABLE II. Precision, recall, F1 score and corresponding standard deviations for the DeepConvLSTM algorithm for the data measured with the professional equipment at the lumbar and the data measured with the left hearing aid with data of different walking conditions incorporated (even/uneven surface without/with head movement).

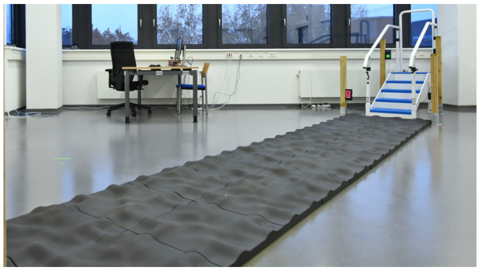

Fig. 1. Track for the "uneven" walking condition with structured surface plates and an additional staircase element.

movement and in the second trial with head movement. For the head movement the participants were instructed to constantly rotate their head between the left and right side in a fast pace, that is still comfortable and feels safe.

Table II shows the performance of the DeepConvLSTM algorithm when the data from "uneven" and "even" walking with and without head movement is incorporated in the "gait trial" data, additionally to the treadmill data. Since the head movement conditions are expected to influence especially data measured at the head most, analysis for the everyday wearable technologies was limited to the left hearing aid. For comparison, also data measured with professional equipment at the lumbar position, as a standard position, is analyzed.

It is noticeable that the performance for the data from all walking conditions and both positions is quite similar. The F1 score shows a value of around 0.83, the recall yields a value of around 0.92, and the precision is around 0.76. A two-way repeated measures ANOVA was conducted to evaluate the effect for different walking conditions on the F1 scores. Mauchly's test of sphericity indicated that the assumption of sphericity was met. The main effect of different walking conditions was not significant ($p=0.741$, partial $\eta^2=0.052$), indicating that incorporating different walking conditions did not change performance. No significant difference was found in the performance of the algorithm for the data measured at the lumbar level and the left hearing aid, regardless of whether the data from the additional walking conditions were incorporated. This suggests that the data collected for the "even" and "uneven" walking conditions, which involved structured surface plates and a staircase element, are sufficiently distinct from the data reflecting participants' responses to perturbations for the algorithm to differentiate between them effectively. This distinction may be explained by the observation that participants' reactions to perturbations typically involve abrupt, irregular movements, which are absent in the additional walking conditions. These results indicate that everyday situations, such as walking over uneven surfaces (e.g. pebbles) or climbing stairs, may not interfere with the detection of gait perturbations.

It might have been anticipated that the additional head movements in the walking conditions would affect especially the algorithm's performance when processing data from the left hearing aid. However, these head movements followed a mostly regular pattern, in contrast to the abrupt and irregular signals caused by the perturbations. Moreover, due to the design of the study the head movements predominantly influence the angular velocity around the vertical axis, while the other two axes and the acceleration data remain largely unaffected. This could explain why the performance of the algorithm does not differ significantly despite the head movements.

Although these findings are a promising starting point, it is important to acknowledge certain limitations. The additional walking conditions included only approximately 1300 data windows per condition, compared to about 25700 windows for treadmill walking. Furthermore, although the additional walking conditions are more realistic than treadmill walking, they were still conducted under controlled laboratory conditions. In real-life walking scenarios, spontaneous and irregular head movements are to be expected, in contrast to the investigated head movements. In particular, real-world head motion often involves substantial pitch and roll components, such as when navigating uneven terrain or avoiding obstacles. However, analyses using only acceleration data, which are less sensitive to rotational movements, yielded comparable performance than using a combination of acceleration and gyroscope data (see section II. Results. C. Performance for acceleration data only; in the main manuscript). This suggests that the algorithm may be robust against complex head movements. Future studies should aim to collect a larger and more balanced dataset for additional walking

conditions to ensure more robust and generalizable results. Additionally, future studies need to investigate the impact of real-life gait data as well as real-life perturbation data on the algorithm's performance to better assess its applicability in everyday settings.

B. Different training and test data

Since there is already data existent for near-falls that was recorded with inertial measurement units at the lumbar or sternum [3]–[7], it would be an advantage if this data could also be used to train the algorithm for the detection with everyday wearable technology. This raises the question of how well the model performs when trained on data from professional devices and tested on data from everyday wearable technologies at different body positions, particularly in the absence of prior preprocessing.

Only the combinations of training data from professional devices at standard positions (lumbar or sternum) and test data from everyday wearable technologies at their typical wearing positions, which showed a correlation coefficient higher than 0.80 in previous analyses (see [8]), were analyzed. These combinations are expected to yield the most promising results, due to the high similarity of the data. This led to the following combinations. For the first combination the algorithm was trained using data recorded at the lumbar level and tested using data obtained with the left hearing aid. For the second and third combination the algorithm was trained using data collected at the sternum level and tested using data collected with the left hearing aid or data collected with a smartphone in a jacket pocket. The resulting performance measures are depicted in Table III. Normality of the F1 scores for each training and test combination was evaluated using the Shapiro-Wilk test, which indicated that the assumption of normality was met. To evaluate statistical significance, a one-way repeated measures ANOVA was performed. Mauchly's test of sphericity indicated that the assumption of sphericity was met. The analysis showed a significant effect of training and test data combination on the F1 score (p<0.001, partial η^2 =0.909). Post hoc comparisons were performed using Bonferroni-adjusted pairwise tests. The F1 scores around 0.40 for different test and training data are significantly lower than all F1 scores obtained when training and testing the algorithm on data from the same device and position, each of which was at least 0.68 (see Table I in the main manuscript). Additionally the F1 score of 0.44, which was retrieved for the combination of sternum and jacket pocket data, was significantly lower than the one retrieved for the combination of sternum or lumbar data with left hearing aid data (about 0.67). It is also noticeable, that the recall does not significantly differ from the recall retrieved from training and testing with data from the same configuration, while the precision shows significantly lower values.

As expected, the performance when training and testing the model with data from different sensor positions is lower than when using data from the same position. However, the results show potential, with a high recall of at least 0.8. As mentioned before, high recall is desirable for long-term monitoring of fall risk as it ensures that most gait perturbations are detected, which is crucial for early detection of changes in the subject's response to disturbances over time. The low precision, recall and F1 score indicate that models trained on data recorded at standard positions such as the sternum or lumbar level, already available from previous studies [3], [9], do not generalize to everyday wearable device placements like hearing aids or smartphones. This suggests that simply transferring models across sensor positions is not feasible without additional processing. The results highlight the necessity of preprocessing or data transformation steps (e.g., coordinate alignment or biomechanical modeling with a mass-spring-damper system), especially given the substantial differences in acceleration and angular velocity signals between positions (e.g., trunk vs. ear). Future studies should focus on developing and validating robust transformation or alignment techniques to further investigate cross-position model generalization.

TABLE III. Precision, recall, F1 score for the DeepConvLSTM algorithm for training with data from professional equipment at lumbar or sternum position and testing with data from everyday wearable technology (LHA: left hearing aid, JP: smartphone in a jacket pocket). Performance metrics (mean ± standard deviation) were calculated on test sets from 10 random participant-level splits.

trained with	tested with	precision	recall	F1
Lumbar	LHA	0.40 ± 0.08	0.36 ± 0.05	0.38 ± 0.05
Sternum	LHA	0.39 ± 0.08	0.51 ± 0.08	0.43 ± 0.07
Sternum	JP	0.40 ± 0.13	0.46 ± 0.17	0.40 ± 0.09

C. Model performance for different perturbation types

The performance results across different sensor positions in the main manuscript (see Table I in the main manuscript) were further analyzed to evaluate performance separately for each individual perturbation type. To provide context for the model's performance, the different types of perturbations applied during treadmill walking are first described in detail. The nine different perturbations were designed to integrate unexpected gait perturbations in various directions:

- Acceleration (right/left): Right/left treadmill belt was accelerated to 180% of the individual's walking speed for about 0.42 s at 3 m/s^2, while the opposite belt maintained a constant speed.
- Deceleration (right/left): Right/left treadmill belt was decelerated to 40% of the individual's walking speed for about 0.42 s at 3 m/s^2, with the opposite belt remaining constant.
- Full stop: Both belts were decelerated to 0 m/s at 9 m/s^2 and held for 0.12 s.
- Sway (right/left): The treadmill was laterally displaced by 5 cm to the right or left at 3 m/s^2.
- Pitch (positive/negative): The treadmill was tilted by $\pm 5°$ for 1 s.

Table IV shows the average recall per perturbation type and sensor position. Significant differences in recall were analyzed using a two-way repeated measures ANOVA. Mauchly's test indicated that the assumption of sphericity was violated. Therefore, the Huynh-Feldt correction was applied. Since the focus was on the overall effects of perturbation type, only the main effects for this factor are reported. The main effect of perturbation type was significant ($p<0.001$, partial $\eta^2=0.289$). Post hoc pairwise comparisons with Bonferroni correction (see Table V) revealed that recall was significantly lower for the full stop perturbation compared to deceleration left, sway right/left, and negative pitch. The lower recall may be related to the distinct characteristics of the full stop perturbation. As it was a bilateral and abrupt perturbation, it was often described by participants as particularly challenging. This may have led to participant reactions that differed significantly from those seen in response to other perturbations, potentially with greater variability across individuals. Combined with the model being trained to detect perturbations in general, without type-specific tuning, this may have reduced classification performance. The analyses also revealed that the perturbation types sway left, sway right, and negative pitch yielded significantly higher recall than most other perturbation types. This finding is notable because these perturbations were subjectively rated among the least challenging by participants. It was initially anticipated that sway and pitch perturbations would be more difficult to detect, as they frequently elicited minimal or no observable reactions in some participants, potentially leading to mislabeling of perturbation windows. However, contrary to this expectation, these perturbations showed the highest detection rates. A likely explanation is that these perturbations were easier to compensate for, leading to more consistent and uniform responses across participants. This similarity in reaction patterns may contributed to the high detection performance. Additionally the mechanical movement of the treadmill, such as lateral displacement during sway, may have further supported detection. To enhance generalizability, future studies should introduce gait perturbations in more ecologically valid settings that minimize such mechanical influences. Additionally, it will be important to investigate older populations with an elevated risk of falling, as their responses may differ considerably from those of younger participants.

Position	accelera-tion right	accelera-tion left	decelera-tion right	decelera-tion left	sway left	sway right	negative pitch	positive pitch	full stop
Lumbar	0.91 ± 0.07	0.95 ± 0.03	0.9 ± 0.07	0.94 ± 0.05	0.95 ± 0.04	0.91 ± 0.07	0.97 ± 0.03	0.88 ± 0.06	0.91 ± 0.05
Sternum	0.88 ± 0.06	0.85 ± 0.07	0.89 ± 0.07	0.92 ± 0.06	0.95 ± 0.7	0.95 ± 0.04	0.93 ± 0.03	0.92 ± 0.05	0.87 ± 0.07
RHA	0.87 ± 0.08	0.88 ± 0.10	0.88 ± 0.09	0.89 ± 0.08	0.94 ± 0.03	0.98 ± 0.04	0.9 ± 0.04	0.88 ± 0.09	0.93 ± 0.06
LHA	0.93 ± 0.07	0.92 ± 0.08	0.93 ± 0.08	0.93 ± 0.06	0.94 ± 0.05	0.97 ± 0.03	0.94 ± 0.07	0.91 ± 0.07	0.87 ± 0.14
JP	0.78 ± 0.08	0.82 ± 0.07	0.83 ± 0.09	0.91 ± 0.06	0.89 ± 0.11	0.92 ± 0.06	0.89 ± 0.07	0.86 ± 0.08	0.80 ± 0.06
PP	0.85 ± 0.09	0.82 ± 0.11	0.88 ± 0.04	0.84 ± 0.08	0.98 ± 0.03	0.97 ± 0.03	0.93 ± 0.07	0.93 ± 0.05	0.81 ± 0.04
SB	0.88 ± 0.06	0.93 ± 0.04	0.80 ± 0.07	0.85 ± 0.08	0.90 ± 0.06	0.97 ± 0.03	0.94 ± 0.06	0.85 ± 0.09	0.77 ± 0.1
Total	0.88 ± 0.09	0.90 ± 0.09	0.87 ± 0.09	0.89 ± 0.07	0.92 ± 0.08	0.94 ± 0.07	0.92 ± 0.07	0.88 ± 0.07	0.87 ± 0.1

TABLE IV. Average recall and corresponding standard deviations for all perturbation types using the DeepConvLSTM algorithm on data measured at different sensor positions. All results are based on the same test runs used for Table I in the main manuscript.

III. Statistical details - Comparison of sensor positions

This section provides additional statistical details corresponding to the sensor position comparisons reported in Table I of the main manuscript. Statistical significance for the F1 scores was assessed using a one-way repeated measures ANOVA with sensor position as the within-subject factor. Since Mauchly's test revealed a violation of sphericity, the Huynh-Feldt correction was applied. Post hoc comparisons were conducted using Bonferroni-adjusted pairwise tests. Table VI reports statistically significant pairwise differences between sensor positions.

References

[1] S. Hellmers, E. Krey, A. Gashi, J. Koschate, L. Schmidt, T. Stuckenschneider, A. Hein, T. Zieschang, "Comparison of machine learning approaches for near-fall detection with motion sensors," Front. Digit. Health, vol. 5, p. 1223845, 2023.
[2] T. T. Um, F. M. J. Pfister, D. Pichler, S. Endo, M. Lang, S. Hirche, U. Fietzek, D. Kulić, "Data augmentation of wearable sensor data for Parkinson's disease monitoring using convolutional neural networks,"Proceedings of the 19th ACM International Conference on Multimodal Interaction. ICMI '17. Glasgow, UK: Association for Computing Machinery, pp. 216-220, 2017.
[3] I. Pang, Y. Okubo, D. Sturnieks, S. R. Lord, M. A. Brodie, "Detection of near falls using wearable devices: a systematic review," J. Geriatr. Phys. Ther., vol. 42, no. 1, pp. 48–56, 2019.
[4] A. Choi, T. H. Kim, O. Yuhai, K. Kim, H. Kim, J. H. Mun, "Deep learning-based near-fall detection algorithm for fall risk monitoring system using a single inertial measurement unit," IEEE Trans. Neural Syst. Rehabil. Eng., vol. 30, pp. 2385–2394, 2022.
[5] M. Trkov, K. Chen, J. Yi, T. Liu, "Inertial sensor-based slip detection in human walking," IEEE Trans. Autom. Sci. Eng., vol. 16, no. 3, pp. 1399–1411, 2019.
[6] S. Wang, F. Miranda, Y. Wang, R. Rasheed, T. Bhatt, "Near-fall detection in unexpected slips during over-ground locomotion with body-worn sensors among older adults," Sensors, vol. 22, no. 9, pp. 3334, 2022.
[7] A. Weiss, I. Shimkin, N. Giladi, J. M. Hausdorff, "Automated detection of near falls: algorithm development and preliminary results," BMC Res. Notes, vol. 3, pp. 1–8, 2010.
[8] L. Feld, L. Schell-Majoor, S. Hellmers, J. Koschate, A. Hein, T. Zieschang, B. Kollmeier, "Comparison of professional and everyday wearable technology at different body positions in terms of recording gait perturbations," PLOS Digital Health, vol. 3, no. 8, 2024.
[9] S. Handelzalts, N. B. Alexander, N. Mastruserio, L. V. Nyquist, D. M. Strasburg, L. V. Ojeda, "Detection of real-world trips in at-fall risk community dwelling older adults using wearable sensors," Front. Med., vol. 7, p. 514, 2020.

P(I)	P(J)	Mean difference (I-J)	p value	P(I)	P(J)	Mean difference (I-J)	p value
acceleration right	acceleration left	-.01	1	acceleration left	acceleration right	.01	1
	deceleration right	-.004	1		deceleration right	.01	1
	deceleration left	-.03	.24		deceleration left	-.02	1
	sway left	-.066*	<.001		sway left	-.054*	<.001
	sway right	-.084*	<.001		sway right	-.071*	<.001
	negative pitch	-.060*	<.001		negative pitch	-.47*	<.001
	positive pitch	-.02	.83		positive pitch	-.01	1
	full stop	.02	1		full stop	.03	.34
deceleration right	acceleration right	.004	1	deceleration left	acceleration right	.03	.24
	acceleration left	-.01	1		acceleration left	.02	1
	deceleration left	-.02	.33		deceleration right	.02	.33
	sway left	-.062*	<.001		sway left	-.39*	.003
	sway right	-.079*	<.001		sway right	-.056*	<.001
	negative pitch	-.056*	<.001		negative pitch	-.032*	.004
	positive pitch	-.02	1		positive pitch	.01	1
	full stop	.02	1		full stop	.045*	.009
sway left	acceleration right	.066*	<.001	sway right	acceleration right	.084*	<.001
	acceleration left	.054*	<.001		acceleration left	.071*	<.001
	deceleration right	.062*	<.001		deceleration right	.079*	<.001
	deceleration left	.039*	.003		deceleration left	.056*	<.001
	sway right	-.02	.46		sway left	.02	.46
	negative pitch	.01	1		negative pitch	.024*	.018
	positive pitch	.045*	<.001		positive pitch	.063*	<.001
	full stop	.083*	<.001		full stop	.10*	<.001
negative pitch	acceleration right	.060*	<.001	positive pitch	acceleration right	.02	.83
	acceleration left	.047*	<.001		acceleration left	.01	1
	deceleration right	.056*	<.001		deceleration right	.02	1
	deceleration left	.032*	.004		deceleration left	-.01	1
	sway left	-.01	1		sway left	-.045*	<.001
	sway right	-.024*	.018		sway right	-.063*	<.001
	positive pitch	.039*	<.001		negative pitch	-.039*	<.001
	full stop	.077*	<.001		full stop	.04	.06
full stop	acceleration right	-.02	1				
	acceleration left	-.03	.34				
	deceleration right	-.02	1				
	deceleration left	-.045*	.009				
	sway left	-.083*	<.001				
	sway right	-.10*	<.001				
	negative pitch	-.077*	<.001				
	positive pitch	-.04	.06				

TABLE V. Statistical comparison with mean difference (I-J) and p value for the recall across perturbation types shown in Table IV. Significant differences in recall were assessed using a two-way repeated measures ANOVA.

Position(I)	Position(J)	Mean Difference (I-J)	p value	Position(I)	Position(J)	Mean Difference (I-J)	p value
Lumbar	Sternum	0.06	0.81	JP	Lumbar	-.11*	0.023
	RHA	-.051	0.053		Sternum	-0.05	1
	LHA	-0.04	0.27		RHA	-.17*	<0.001
	JP	.11*	0.023		LHA	-.16*	0.002
	PP	.12*	0.003		PP	0.01	1
	SB	0.11	0.28		SB	0	1
Sternum	Lumbar	-0.06	0.81	PP	Lumbar	-.12*	0.003
	RHA	-.11*	0.027		Sternum	-0.06	1
	LHA	-.11*	0.041		RHA	-.17*	0.001
	JP	0.05	1		LHA	-.17*	<0.001
	PP	0.06	1		JP	-0.01	1
	SB	0.05	1		SB	-0.01	1
RHA	Lumbar	.051	0.053	SB	Lumbar	-0.11	0.28
	Sternum	.11*	0.027		Sternum	-0.05	1
	LHA	0.01	1		RHA	-.16	0.063
	JP	.17*	<0.001		LHA	-.16*	0.013
	PP	.17*	0.001		JP	0	1
	SB	.16	0.063		PP	0.01	1
LHA	Lumbar	0.027	0.08				
	Sternum	.11*	0.041				
	RHA	-0.01	1				
	JP	.16*	0.002				
	PP	.17*	<0.001				
	SB	.16*	0.013				

TABLE VI. Summary of statistical test results comparing the F1 scores between sensor positions (lumbar, sternum, left/right hearing aid (LHA/RHA), smartphone in a jacket pocket/pants pocket/shoulder bag (JP/PP/SB)) displayed in Table I in the main manuscript. (*) denote significant differences ($p \leq 0.05$). Statistical significance for the F1 scores was tested using a one-way repeated measures ANOVA.
